# IPD-Net: Infrared Pedestrian Detection Network via Adaptive Feature Extraction and Coordinate Information Fusion

**DOI:** 10.3390/s22228966

**Published:** 2022-11-19

**Authors:** Lun Zhou, Song Gao, Simin Wang, Hengsheng Zhang, Ruochen Liu, Jiaming Liu

**Affiliations:** 1 Key Laboratory of Earth Exploration and Information Techniques (Chengdu University of Technology), Ministry of Education, Chengdu 610059, China; 2 The College of Mechanical and Electrical Engineering, Chengdu University of Technology, Chengdu 610059, China

**Keywords:** infrared images, infrared target detection, pedestrian detection, attention mechanism, YOLOv5

## Abstract

Infrared pedestrian detection has important theoretical research value and a wide range of application scenarios. Because of its special imaging method, infrared images can be used for pedestrian detection at night and in severe weather conditions. However, the lack of pedestrian feature information in infrared images and the small scale of pedestrian objects makes it difficult for detection networks to extract feature information and accurately detect small-scale pedestrians. To address these issues, this paper proposes an infrared pedestrian detection network based on YOLOv5, named IPD-Net. Firstly, an adaptive feature extraction module (AFEM) is designed in the backbone network section, in which a residual structure with stepwise selective kernel was included to enable the model to better extract feature information under different sizes of the receptive field. Secondly, a coordinate attention feature pyramid network (CA-FPN) is designed to enhance the deep feature map with location information through the coordinate attention module, so that the network gains better capability of object localization. Finally, shallow information is introduced into the feature fusion network to improve the detection accuracy of weak and small objects. Experimental results on the large infrared image dataset ZUT show that the mean Average Precision (mAP50) of our model is improved by 3.6% compared to that of YOLOv5s. In addition, IPD-Net shows various degrees of accuracy improvement compared to other excellent methods.

## 1. Introduction

**Figure 1:**
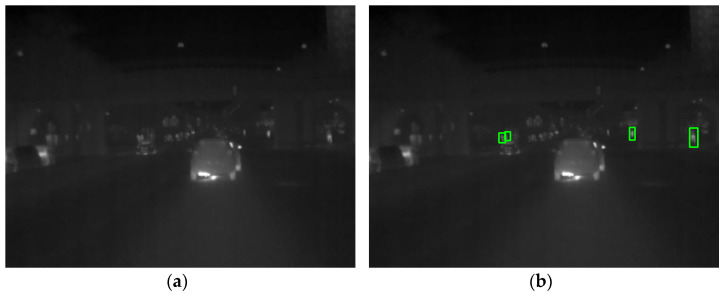
Infrared image instance: (**a**) original infrared image; (**b**) infrared image with annotations.

Pedestrian detection is an important research direction in the field of object detection, with a wide range of applications in automotive assisted driving systems [1,2], intelligent transportation [3], and video surveillance [4,5]. Therefore, it has received a lot of attention from researchers in the field of computer vision. With the rapid development of computer vision, machine learning, and deep learning techniques, pedestrian detection techniques based on infrared images are also advancing. Infrared device imaging relies on the thermal radiation of an object. The higher the heat radiated by the object, the brighter it appears on the infrared image [6]. Thus, infrared equipment imaging is less influenced by lighting and weather conditions and is more adaptable to the environment. It still works well in low light and even in complete darkness. In addition, infrared pedestrian detection systems are also more resistant to interference and can overcome visual barriers in severe weather conditions to achieve good detection results and a wider range of applications and time periods. However, pedestrians are often blended with the background in infrared images as the image quality of infrared devices is strongly influenced by temperature, which leads to difficulties in detecting pedestrians in infrared images [7]. In addition, the special imaging method of infrared devices results in images with little texture detail, low signal-to-noise ratio, and weak contrast, causing poor pedestrian detection in infrared images [8,9]. An instance of the infrared pedestrian is shown in Figure 1. The original image is shown in Figure 1a, and the green boxes in Figure 1b are the pedestrian annotations in the dataset.

In recent years, researchers have proposed many object detection algorithms that have been applied to the field of infrared image pedestrian detection with some achievements. In 2014, Girshick et al. [10] proposed R-CNN, which was the first time use convolutional neural network (CNN) for object detection. Since then, many CNN-based two-stage detection algorithms have been proposed, and the accuracy and speed of object detection have been substantially improved. In 2015, Redmon et al. [11] proposed YOLO object detection, which offers a significant speed-up compared to the two-stage object detection algorithms. As the pioneer of one-stage object detection algorithms, YOLO still has some shortcomings, but has been followed by a series of better algorithms such as YOLO9000 [12], YOLOv3 [13], YOLOv4 [14], and YOLOv5 [15]. As an advanced one-stage object detection algorithm released in 2020, YOLOv5 offers four different-sized models for easy selection in industrial deployments. Since pedestrian detection in infrared images is mostly applied to moveable devices, we chose the less complex YOLOv5s model as the baseline.

**Figure 2:**
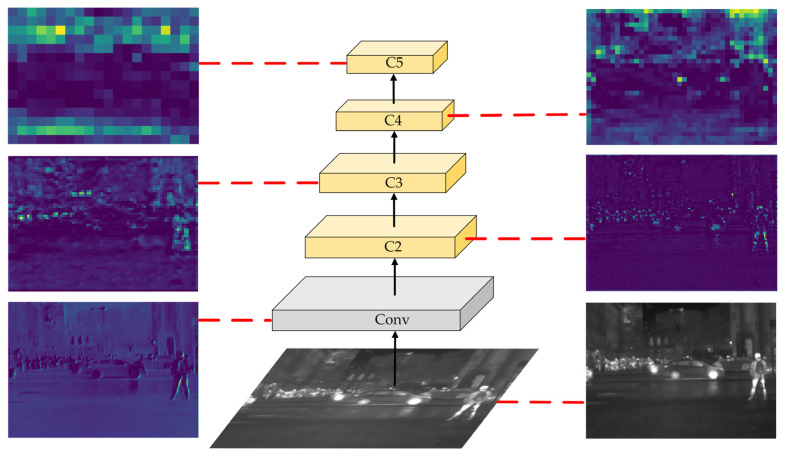
Feature heat maps in YOLOv5s backbone network.

YOLOv5s extracts the feature information of pedestrians in infrared images through the backbone network and obtains feature maps at different scales for prediction after the feature fusion network. Figure 2 shows the feature maps of each layer extracted by the YOLOv5s backbone network and the corresponding heat map of each layer. For pedestrian objects in infrared images that are highly similar to the background and have weak features, the feature extraction capability of the YOLOv5s backbone network is insufficient, resulting in less feature information being extracted, as shown in the heat map corresponding to the feature maps in layers C2 and C3. In addition, as the network structure deepens, pedestrian object information with weaker features and smaller sizes is gradually lost, as shown in the corresponding heat maps of C4 and C5. That weak and small object information is mainly concentrated in the shallow feature maps C2 and C3. The feature fusion network of YOLOv5s only uses the feature maps of C3, C4, and C5 for feature fusion, failing to make full use of the small object information contained in C2, resulting in low accuracy in detecting weak and small objects in infrared images. Therefore, to address the problems of YOLOv5s in infrared image pedestrian detection, this paper proposes a more suitable algorithm for infrared image pedestrian detection, IPD-Net.

The main contributions of the research can be summarized as follows:

1. In the backbone, an adaptive feature extraction module (AFEM) is designed to extract pedestrian features. By introducing an improved selective kernel attention module into the residual structure, the AFEM obtains a multi-scale receptive field to better distinguish the object from the background and obtains better pedestrian feature information extraction capability.
2. In the neck, this paper designs a coordinate attention feature pyramid network (CA-FPN). Firstly, aiming at small and weak objects present in the infrared images, we introduced the feature maps of the C2 layer into the feature fusion network, making full use of the small and weak object information contained in the feature maps of the C2 layer. In addition, a coordinate attention module is introduced to encode the feature map position information in layers C3, C4, and C5 to enhance the position information of the objects in the feature map. The fusion by concatenating results in a better balance of positional and semantic information contained in each layer of the feature map, improves the feature representation capability of the network.
3. In the head, we propose a new bounding box loss function α-EIoU, which improves the bounding box regression capability of the model, speeds up the convergence of the model, and obtains a better object localization capability.
4. This paper analyses the problems of pedestrian objects in infrared images, including the lack of feature information and the small scale of pedestrian objects, and proposes an improved infrared pedestrian detection model, IPD-Net, based on YOLOv5s. Through validation on the Zachodniopomorski Uniwersytet Technologiczny (ZUT) dataset [16], IPD-Net achieves higher detection accuracy compared with some current mainstream detection networks.

## 2. Related Work

Pedestrian detection in infrared images has a robust environmental adaptation and anti-interference capability. Therefore, infrared image pedestrian detection has become a popular research topic in the field of object detection, and researchers have conducted a lot of research in this direction. Currently, there are two main categories of pedestrian detection in infrared images: traditional detection methods and deep learning-based detection methods.

Traditional methods rely mainly on handcrafted features for pedestrian detection. Dala et al. [17] proposed a pedestrian detection algorithm based on a Histogram of Oriented Gradient (HOG) combined with a Support Vector Machine (SVM). HOG divides the image into basic cells of the same size, collects the gradient direction density distribution of each pixel in each cell to represent the shape and features of the pedestrian, and then trains the SVM to achieve classification. The HOG approach is robust to changes in light and scale. Zhang et al. [18] proposed an improved pedestrian detection method with Haar-like features, which incorporates daily knowledge into a simple and computationally efficient feature design and achieves better performance at low computational performance. Brenhar et al. [19] performed fast Region of Interests (ROIs) extraction for more rapid detection using the higher luminance of pedestrian heads and legs in infrared images. A faster detection speed was obtained by fusing four features of the infrared pedestrian detection algorithm with HOG features, the Local Binary Pattern (LBP) [20], normalized gradient amplitude, and brightness channel. Traditional pedestrian detection algorithms are mainly based on manually designed feature extraction procedures to achieve pedestrian detection, which are cumbersome to implement and have weak generalization capabilities.

**Figure 3:**
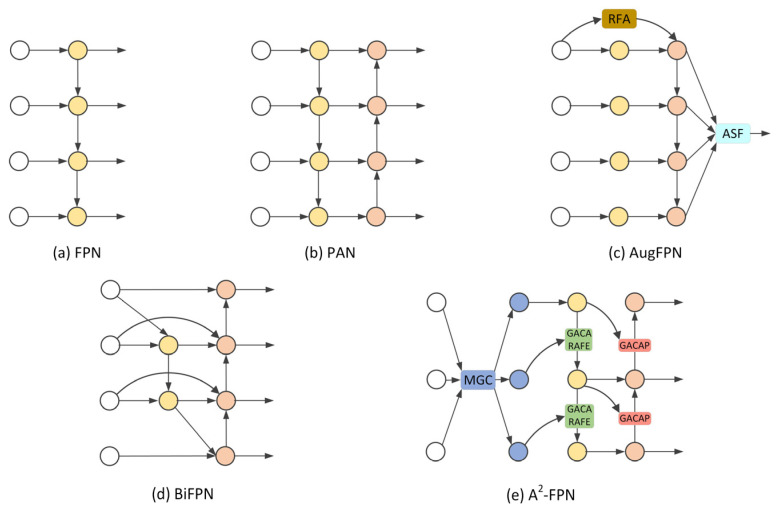
Feature Pyramid Network and its variants.

To solve these problems, object detection based on deep learning is used in the field of infrared pedestrian detection. Compared with traditional detection algorithms, the deep learning approach detects pedestrian features by learning them from a large number of images. In 2014, Girshick proposed a deep neural network approach to object detection, which substantially improved the performance of object detection by using multi-layer convolution networks to extract more abstract semantic information. To fully fuse the feature information extracted by CNN, Lin et al. [21] proposed a top-down structure with lateral connections, known as a Feature Pyramid Network (FPN). Subsequently, a large number of FPN variants were devised. Li et al. [22] proposed the Path Aggregation Network (PANet), which adds bottom-up paths to enhance the feature representation capability of the feature fusion network. Guo et al. [23] proposed the Augmented FPN (AugFPN) consisting of Consistent Supervision, Residual Feature Augmentation, and Soft RoI Selection components to solve the information loss problem of the feature fusion network. Tan et al. [24] designed a weighted Bi-directional Feature Pyramid Network (BiFPN) for fast and efficient feature fusion. Hu et al. [25] proposed an Attention Aggregation-based Feature Pyramid Network (A^2^-FPN), which enhances semantic consistency and obtains better fusion by aggregating complementary information of adjacent features and channel weighting. FPN and its variants enhance feature fusion through lateral connectivity, top-down and bottom-up information transfer, and channel weighting, ultimately improving object detection performance. Figure 3 shows the basic structures of the FPN and its variants.

FPN uses the feature maps extracted by the backbone network to achieve the fusion of adjacent features and strong semantic information through a lateral connection and top-down pathway, as shown in Figure 3a. Based on FPN, PAN adds an extra bottom-up pathway, as shown in Figure 3b, shortening the path from the bottom to the top of the feature map and enhancing the fusion of information in shallow feature maps. As shown in Figure 3c, AugFPN adds the Residual Feature Augmentation (RFA) and Adaptive Spatial Fusion (ASF) modules, which mainly address the information loss during feature fusion. The BiFPN fuses the feature maps extracted from the backbone network more fully by using lateral skip connections, as shown in Figure 3d, without adding too much computational cost. As shown in Figure 3e, A^2^-FPN uses Multi-level Global Context (MGC) to extract more discriminative features, the Global Attention CARAFE (GACARAFE) module for top-down path enhancement, and the Global Attention Content-Aware Pooling (GACAP) module for bottom-up path enhancement.

In addition to the above methods, researchers have made many pedestrian detection algorithms in the field of infrared pedestrian detection. Qu et al. [26] proposed an algorithm for infrared pedestrian detection based on Faster R-CNN [27] with an improved two-layer Region Proposal Network (RPN). By designing a two-layer RPN pyramid and introducing the Inception-v4 module [28], the network enables the capture of richer multi-scale information, uses the PSalign pooling to extract local features of the object, and fully exploits the foreground information of the image. Li et al. [29] proposed the SE-YOLOv3 infrared pedestrian detection algorithm, which introduced the Squeeze-and-Excitation (SE) module [30] into YOLOv3 to improve the feature description capability of the network and obtain a good result on small-scale pedestrian detection. Wang et al. [31] proposed an infrared pedestrian detection network PPDet based on deep learning, which had the strong capability of pixel-by-pixel prediction. By designing a Shortcut-Dilation Network (SDN), a Multi-Scale Feature Enhancement Module (MSFEM), and a multiple cascaded Pixel-by-Pixel Prediction Head (PPPH), this network obtained better capabilities of feature extraction, feature fusion, and head prediction. Yu et al. [32] designed an improved infrared pedestrian detection algorithm based on YOLOv3, which enhances the feature extraction capability of the network by adding an Efficient Channel Attention (ECA) module [33] and an improved Cross-layer Spatial Pyramid Pooling module (CSPP) to the backbone network. Dai et al. [34] proposed a region-free object detection framework named TIRNet, similar to SSD, which can learn the more discriminative and robust features by a Custom SSD (CSSD) and Residual Branching (RB). Li et al. [35] proposed an area-free object detector for infrared images based on YOLOv5, called YOLO-FIRI, which obtains good infrared pedestrian detection performance by improving the CSP structure, introducing multiple detection heads, and enhancing the images.

These methods have been adapted for pedestrian detection in infrared images by modifying existing object detection algorithms, but they still have some shortcomings. Firstly, the size of the pedestrian target in the infrared image differs considerably depending on the distance, and fixed convolution kernels cannot adapt better to changes in the target size. Secondly, there are many pedestrian objects with weak features and small scales in the infrared images, and information about those objects is lost as the network deepens. Therefore, pedestrian detection in infrared images requires effective feature fusion networks to fuse feature maps of different resolutions to enhance feature representation. Finally, these algorithms’ bounding box loss functions need to be better adapted to the bounding regression of pedestrian targets in infrared images. Thus, we propose an improved infrared pedestrian detection network, IPD-Net, based on YOLOv5s. IPD-Net has an adaptive feature extraction module, AFEM, which adaptively adjusts the convolutional kernel receptive field to provide better feature extraction for pedestrians at different scales. In addition, the CA-FPN we designed introduces shallow information into the feature fusion network and fully exploits the localization information of targets in the deep feature map, solving the problem of missing information of weak and small targets and the lack of localization information in the feature map of the deep network. Finally, IPD-Net uses an improved bounding box loss function called α-EIoU, which is more suitable for pedestrian detection in infrared images.

## 3. Proposed Method

**Figure 4:**
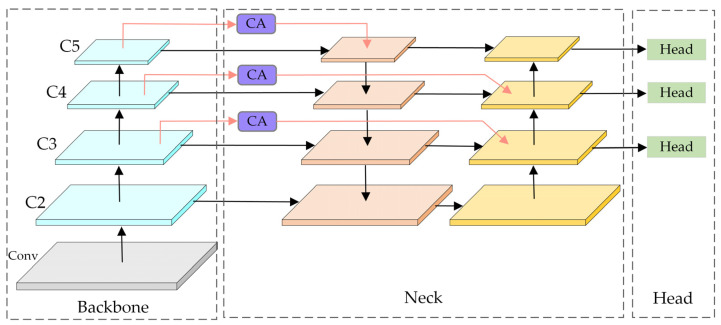
The overall structure of IPD-Net.

The overall structure of the infrared pedestrian detection network proposed is shown in Figure 4. After the raw infrared images are input to the network, they first carry out feature extraction through a backbone network composed of AFEM to obtain a pyramid of feature maps in different scales. Then, the feature map is fused with features through our designed feature fusion network CA-FPN, which can make the information in each layer more balanced and enhance the feature representation at different levels. At last, the result detection at different scales is achieved through three detection heads.

### 3.1. Backbone

**Figure 5:**
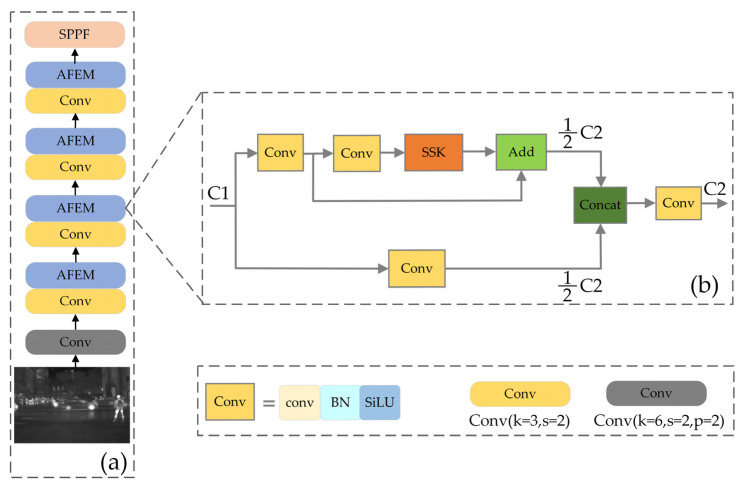
The specific structure of backbone and AFEM block: (**a**) structure of the IPD-Net backbone; (**b**) structure of the AFEM.

The backbone network is mainly used to extract the feature information of pedestrians in infrared images. As shown in Figure 5a, the backbone network of IPD-Net consists of a stack of Conv and AFEM. The Conv contains three operations, standard convolution, normalization, and activation functions. The structure of the AFEM module is shown in Figure 5b, where the input feature maps are operated in two separate ways. The feature map of one path is first passed through convolutions to adjust the number of channels to 0.5 times C2 and then through a residual structure consisting of an SSK module. Another way adjusts to 0.5 times the number of channels of C2 by a convolution. Then the two parts are concatenated to obtain the output with the number of channels of C2, and finally the concatenate features are fused using a Conv convolution block. By using the AFEM convolution module, a richer combination of gradients can be achieved, the learning capability of the CNN is effectively enhanced, and the computational effort is reduced.

**Figure 6:**
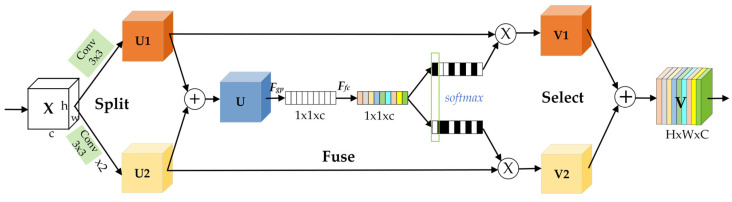
The specific structure of the SSK block.

The SSK is a module based on an improved selective kernel (SK). In standard CNN, it is flawed that the receptive fields of each layer of artificial neurons are designed to have the same scale. Each neuron should be able to adaptively adjust its receptive field size according to the input information, so that convolution kernels with different receptive fields can extract richer feature information [36]. Therefore, an SK model is designed to capture the feature information of objects, which can adaptively adjust the convolution kernel size to 3, 5, and 7. However, introducing larger-scale convolution kernels results in a heavier number of parameters. To address this problem, we designed an SSK block with the structure shown in Figure 6. In a convolutional neural network, two cascaded 3 × 3 convolutional kernels have the same receptive fields as a 5 × 5 convolutional kernel and will consume fewer computation resources [37]. Therefore, we can use two cascaded 3 × 3 convolution kernels in series instead of one 5 × 5 convolution kernel to reduce the computational effort while obtaining the same receptive fields.

The SSK module is shown in Figure 6. Three main operations are carried out in the SSK module: splitting, fusion, and selection. The input feature map X is split into two pathways, passed through a 3 × 3 convolution kernel and two stacked 3 × 3 convolution kernels to obtain feature maps U1 and U2, respectively. Then, U1 and U2 are summed to obtain the fused feature map U. In the fusion stage, U is compressed to 1 × 1 × C by a global average pooling, and the corresponding weight encoding is extracted by the SoftMax function after two full connection layers. Finally, the obtained weight-encoding values are multiplied with U1 and U2, respectively, in the select stage and added together to obtain the feature map V, which contains all weight-encoding information. After splitting, fusion, and selection, the obtained result V incorporates the feature information extracted from the receptive field so that the network adaptively adjusts the receptive field using a similar way to channel attention. Compared with the 3 × 3 convolution kernel in the original residual structure, SSK obtains a multi-scale receptive field and has better feature information extraction capability. It can extract pedestrian feature information from infrared images more effectively and receive feature maps with richer feature information. It solves the problem of YOLOv5s having insufficient ability to extract feature information in infrared images.

### 3.2. Neck

After feature extraction by the backbone network, feature maps with different resolutions are obtained. As the depth of the network varies, the information in the feature maps differs somewhat. Shallow feature maps contain more location and small object information, which is more beneficial for object localization and small-scale object detection than deeper networks. In addition, the feature maps extracted in deeper layers contain more high-layer semantic information than the shallow ones and are more useful for classification. Therefore, an effective feature fusion network is needed to fully fuse the feature map information from the different layers.

#### 3.2.1. Shortcomings of the PAN in YOLOv5s

**Figure 7:**
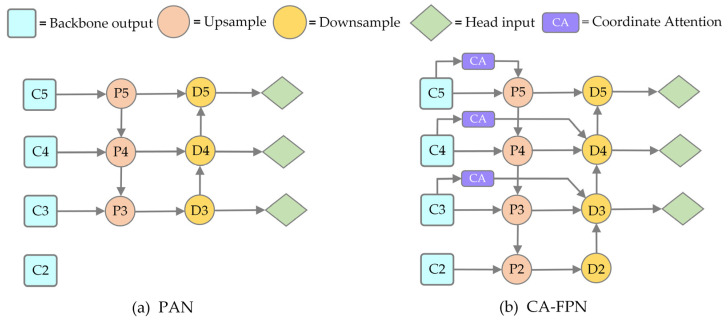
Comparison of feature fusion networks in YOLOv5s and IPD-Net.

YOLOv5s uses the structure of FPN and PAN for the multi-scale fusion of features, as shown in Figure 7a. The FPN structure is upsampled by a top-down method and then fused with each feature map layer through lateral connections to introduce high-level semantic information from the deep feature map into the shallow network. The PAN structure adds bottom-up downsampling to bring the location and small object information from the shallow feature maps into the deeper network. The PAN is structured so that the layers of feature maps contain more balanced information and are more conducive to pedestrian detection by the detection head block. However, the PAN feature fusion network still has two problems: (1) Infrared images contain many weak and small objects. As the number of convolution layers increases, some weak and small objects will be lost, so the PAN network does not make full use of the shallow feature maps for fusion. (2) The PAN network needs to dig deeper into the deep feature maps for localization information. In addition, the PAN structure fuses the location information and small object information in the shallow feature map into the deep feature map by downsampling. Still, a large amount of information is lost in the downsampling process.

To address these two main problems, inspired by PAN and BiFPN, we designed CA-FPN to obtain better feature fusion. The structure of CA-FPN is shown in Figure 7b, which enhances feature reuse by adding lateral skip connections, uses the coordinate attention module to further exploit the localization information of targets in the deep feature map, and achieves full fusion of feature information and location information of weak and small targets.

#### 3.2.2. Enhanced Fusion of Shallow Feature Maps

To solve the problem that the deepening of the YOLOv5 network leads to a missing weak pedestrian object in the feature map, we make full use of the information in the shallow feature maps. As shown in Figure 7b, we added a lateral connection of layer C2 to the feature fusion network and introduced the feature maps of layer C2 to the bottom of the feature fusion network. The P3 layer feature map is upsampled and fused with the C2 layer feature map to obtain P2, which is then downsampled and further fused to obtain the final prediction. The introduction of C2 and P2 layer feature maps enhances the use of weak objects in the shallow feature maps. It improves the model’s detection accuracy for weak and small objects in infrared images.

#### 3.2.3. Feature Fusion with Coordinate Attention Model

**Figure 8:**
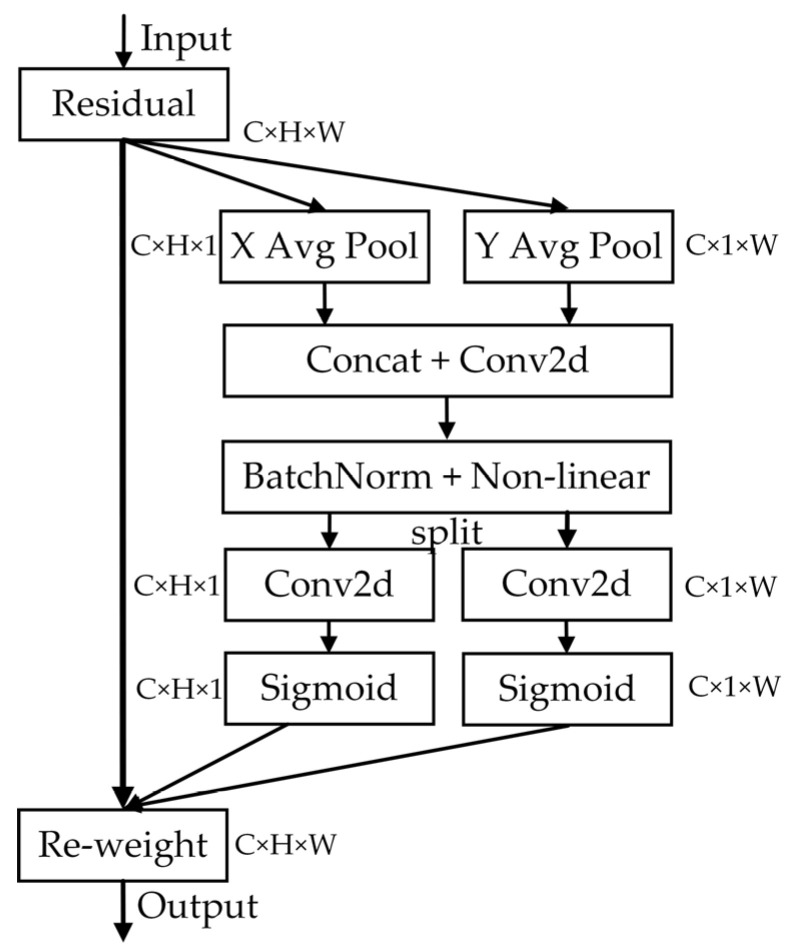
Structure of the Coordinate Attention Module.

We use the coordinate attention (CA) [38] module in CA-FPN to enhance the extraction of location information in the deep network feature maps. The structure of the CA module is shown in Figure 8. After averaging pooling along the *x*-direction (H) and *y*-direction (W), respectively, the CA block extracts weights for both the *x* and *y* directions, respectively, to obtain global location encoding information. Then, the extracted location encoding information fuses with the original feature map to enhance the location information in the feature map.

To fully dig into the deeper feature map location information, we introduced the CA module into the feature fusion network. C3, C4, and C5 feature maps are enhanced with position information by the CA module and multiplexed using skip connections. C3 and C4 are passed through the CA module and then concatenated to obtain D3 and D4 feature maps, and C5 is passed through CA and concatenated to obtain the P5 feature map. The position information in the C3, C4, and C5 feature maps are enhanced by using the coordinate attention module so that the feature maps contain more information on object positioning.

In the CA-FPN, the shallow feature map is first introduced to make full use of the weak and small object information in the shallow feature map and improve the detection accuracy of the model for weak objects. Secondly, the position information in the feature map is encoded by the coordinate attention module to enhance the ability to mine the localization information in the deep feature map, improve the localization capability of the infrared detection model, and enhance the detection accuracy.

### 3.3. Head

The head of YOLOv5s predicts feature maps at different scales after fusion. During the training process, the loss function calculates the loss value between the predicted and real values. The model then adjusts the parameters by back-propagation to gradually reduce the loss value and finally accurately detect pedestrians in infrared images. The loss function in YOLOv5s consists of classification loss, localization loss, and confidence loss. Specifically, classification loss calculates whether the anchor box is correct concerning the corresponding calibration classification, localization loss is the error between the predicted bounding box and the true bounding box, and confidence loss calculates the confidence level of the network. One of the most critical tasks in object detection is bounding box prediction. In pedestrian detection, the pedestrian object needs to be correctly framed by gradually adjusting the position of the predicted bounding box. The bounding box loss function used in YOLOv5s is CIoU loss [39]. CIoU loss is obtained by improving the IoU loss. The three elements (the overlap area between the predicted and real boxes, centroid distance, and aspect ratio bounding box regression) are considered in the CIoU loss function. The CIoU loss function equation is as follows:

(1) $$L_{CIoU}=1-IoU+\frac{\rho^{2}\left(b,b_{gt}\right)}{c^{2}}+\alpha v$$

where v is:

(2) $$v=\frac{4}{\pi^{2}}{\left(\arctan\frac{w_{gt}}{h_{gt}}+\arctan\frac{w}{h}\right)}^{2}$$

c is the diagonal length of the minimum closed box covering the two bounding boxes, b and $b_{gt}$ are the central points of two bounding boxes, and ρ is specified as Euclidean distance.

The CIoU loss function takes the aspect ratio of the bounding box into account. However, in Equation (1), v reflects only the difference between the predicted and true bounding box aspect ratios, not the true relationship between w and $w_{gt}$ (h and $h_{gt}$). While increasing the similarity of aspect ratios, it prevents the model from effectively reducing the true difference between (w,h) and $\left(w_{gt},h_{gt}\right)$. To address this issue, we improve the EIoU loss function [40] and propose a new bounding box loss function named α-EIoU loss, which is defined as follows:

(3) $$L_{\alpha -EIoU}=1-IoU+\alpha_{1}\times \frac{\rho^{2}\left(b,b_{gt}\right)}{c^{2}}+\alpha_{2}\times \frac{\rho^{2}\left(w,w_{gt}\right)}{C_{w}^{2}}+\alpha_{3}\times \frac{\rho^{2}\left(h,h_{gt}\right)}{C_{h}^{2}}$$

This loss function contains three components, IoU loss, distance loss, and aspect ratio loss. $C_{w}$ and $C_{h}$ are the width and height of the minimum closed box covering the two bounding boxes, respectively. The width and height ratio between the predicted bounding box and the real bounding box is defined in the α-EIoU loss function. It solves the problem that the aspect ratio between the predicted bounding box and the real bounding box of the CIoU loss function does not correspond directly. In the actual regression process, the distance between the center of mass of the predicted and the real bounding box more directly reflects the distance relationship between them. It should be given greater weight than the aspect ratio. Therefore, parameters $\alpha_{1}$, $\alpha_{2}$, and $\alpha_{3}$ are added to adjust the weight shares of the center distance, width ratio, and height ratio of the object bounding box in the loss function, respectively, ultimately improving the ability of the bounding box regression. The α-EIoU loss directly minimizes the difference in width and height between the predicted and real bounding box. It can speed up convergence, improve bounding box regression, and improve the accuracy of the model detection head for pedestrian detection in infrared images.

## 4. The Experiment and Result Analysis

To validate the performance of the IPD-Net proposed in this paper, we conducted validation experiments on the ZUT dataset. Firstly, we performed ablation experiments and compared them with the baseline YOLOv5s to validate the impact of improved modules on the performance of IPD-Net. Secondly, comparative experiments on infrared images at different scales verified the performance of the IPD-Net on images at different scales. Finally, we carried out control experiments with other existing algorithms to further validate the performance of IPD-Net.

### 4.1. Experimental Environment and Settings

We used a device with an Intel Xeon Platinum 8260C CPU and NVIDIA GeForce RTX 3090 GPU for our experiments. The input image was 320 × 320, the training rounds 40, the batch size 32, the initial learning rate 0.005, the IoU threshold 0.4, and the momentum and weight decay were 0.937 and 0.0005, respectively. All experiments were trained based on PyTorch 1.10 and python 3.8.

### 4.2. Dataset

We used the ZUT dataset, an infrared image dataset of roads in Poland, Lithuania, Germany, and Denmark with a resolution of 640 × 480. It includes several road scenarios, including the city center, old town, roundabouts, tunnels, city outskirts, one-way roads, two-way roads, highways, and autobahns, and different weather conditions such as sunny weather, cloudy with rain, light rain, heavy rain, and fog. The dataset was annotated with nine categories of labels using the Ybat: YOLO BBox Annotation Tool. During the experiment, we removed the non-pedestrian labels and modified all other labels containing human to “pedestrian”. By processing the dataset like this, we ended up with 32,398 images and a total of 105,702 objects. We then divided the images into a training and validation set in a ratio of 7:3 for the experiments.

### 4.3. Ablation Experiment

To show the impact of each module on our model directly, we conducted ablation experiments in this part. Specifically, we used YOLOv5s as the baseline and added the AFEM, the feature fusion network CA-FPN, and the α-EIoU loss function to the model, respectively. By comparing with the detection results of the baseline, we can evaluate the performance improvement brought by each module. Finally, all the improved modules were added to the network to obtain the impact of their combined effect.

Our ablation experiments were trained for 40 epochs with an input size of 320 × 320, and the results obtained after the training results were finally stabilized are shown in Table 1. The first row of the table shows the results of the baseline YOLOv5s detection. Firstly, we added AFEM, CA-FPN, and α-EIoU to the baseline separately, and the three sets of detection results were obtained. The detection accuracies of the experiments were all higher than the baseline YOLOv5s, indicating that each of our improvement points is useful. Secondly, we conducted three two-by-two comparison experiments. The network with AFEM and CA-FPN showed little change in detection accuracy compared to the network with CA-FPN alone. We consider that the role of AFEM is to find out the location of the target, the role of CA-FPN is to enhance the features of the target, and they both serve to capture the target better. AFEM and CA-FPN focus on identifying the target in the background, and the mechanism is the same for object detection, which may result in little variation in accuracy. In addition, adding the α-EIoU loss function to the networks with AFEM or CA-FPN, respectively, the networks were detected with higher accuracy than adding each improvement alone. This is because α-EIoU can regress the boundary of the target more accurately than YOLOv5s’ CIoU by enhancing the direct correspondence of the bounding box aspect ratio and the calculation of the center distance. Finally, all three improvements were added to the model. AFEM and CA-FPN capture richer feature information about the target, while α-EIoU makes the network achieve better classification and regression. The combination of these three provides more significant improvements in detecting weak and small infrared targets and obtains the highest accuracy.

### 4.4. Experiments with Different Input Scales

We completed experiments on infrared images of different sizes. Expressly, we set the model input image sizes to 320 × 320, 480 × 480, and 640 × 640 to obtain the experimental results of IPD-Net and YOLOv5s, as shown in Table 2. The experimental results show that compared to the YOLOv5s baseline, IPD-Net has different degrees of accuracy improvement at different scales. At a scale of 320 × 320, IPD-Net’s mAP50 improves by 3.6 points compared to YOLOv5s. This proves that our model is more suitable than YOLOv5s for detecting pedestrians in small-scale infrared images and is more beneficial when performing industrial deployments. In addition, on the 480 × 480 and 640 × 640 scales, IPD-Net obtains higher accuracy than YOLOv5s. The results from the experiments conducted on different image scales show that our proposed model IPD-Net is robust to changes in image size.

### 4.5. Comparative Experiments with Different Algorithms

#### 4.5.1. Comparison with YOLO Series Algorithms

To verify the effectiveness and advancement of IPD-Net, we conducted comparative experiments with the current mainstream one-stage object detection algorithms. We set the input image size to 320 × 320, and the experimental results are shown in Table 3. IPD-Net was compared with YOLOv3-tiny, YOLOv4-tiny, YOLOv5s, YOLOX-s [41], and YOLOv7 [42] object detection algorithms for the experiments. The results show that the proposed IPD-Net exhibits the best performance and the highest mAP value, demonstrating its effectiveness compared to other models.

**Figure 9:**
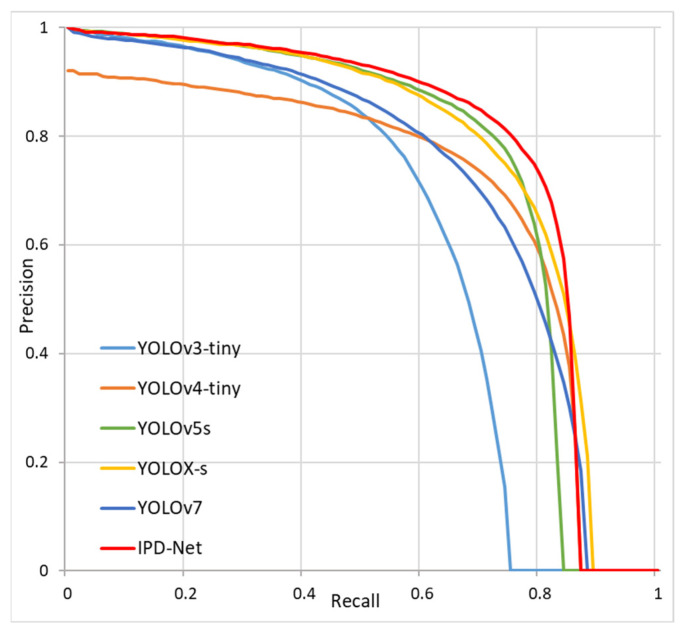
Precision–recall curves for the YOLO series algorithms.

In addition, we plotted precision–recall curves for each model to show more visually how IPD-Net compares to other models. As shown in Figure 9, the model’s performance is compared visually by calculating the area enclosed by the precision–recall curve with the precision and recall axes. The larger the area enclosed by the curve, the better the performance of the model. It is obvious from the graph that IPD-Net, the red curve in Figure 9, has the largest area so that IPD-Net outperforms other lightweight object detection algorithms.

#### 4.5.2. Comparison with Other Object Detection Algorithms

To verify the effectiveness and sophistication of IPD-Net, we conducted experiments comparing IPD-Net with existing object detection methods. Some one-stage and two-stage target detection algorithms were selected for experiments, respectively, and the input image size was set to 640 × 640, and the experimental results are shown in Table 4. Compared to the baseline YOLOv5s, IPD-Net showed a 2.1% increase in mAP50 and a 1.1% increase in mAP(50:95). The mAP50 and mAP(50:95) of IPD-Net are still the highest compared to other methods, demonstrating that IPD-Net has the best pedestrian detection performance for infrared images.

**Figure 10:**
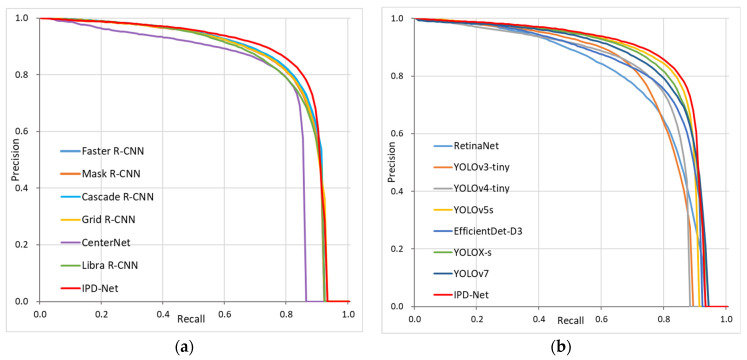
Precision–recall curves for IPD-Net and mainstream target detection algorithms: (**a**) two-stage algorithms; (**b**) one-stage algorithms.

To visually measure the performance of IPD-Net against other existing algorithms, we plotted the precision–recall curves of the two-stage and one-stage algorithms, respectively. Figure 10a shows the precision–recall curves for the IPD-Net and two-stage object detection algorithms, while Figure 10b shows the precision–recall curves for the IPD-Net and one-stage algorithms. As seen in Figure 10, the precision–recall curve for IPD-Net has the largest area enclosed by the axes, verifying that IPD-Net has the best performance.

#### 4.5.3. Visualization of Experimental Results

**Figure 11:**
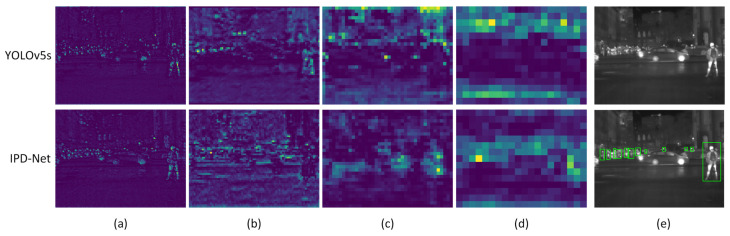
Visualization of the feature heat maps extracted from the backbone networks of IPD-Net and YOLOv5s: (**a**–**d**) represent the feature heat maps of the C2, C3, C4, and C5 layers of the backbone networks, respectively; (**e**) shows the original image and ground truth, and green boxes represent pedestrians annotated in the dataset.

To demonstrate the effectiveness of our proposed AFEM for feature extraction, we visualized and compared the feature maps extracted from the IPD-Net and YOLOv5s backbone networks, as shown in Figure 11. Higher heat levels in the figure indicate that more feature information was extracted from the region. The figure shows that the heat values of the feature heat map extracted by YOLOv5s are more dispersed and hardly concentrated in the pedestrian area. In contrast, the heat values of IPD-Net’s feature heat map are precisely focused on the pedestrian area and have excellent feature extraction for pedestrians of different scales. The results demonstrate that the backbone network of IPD-Net has better feature extraction capability.

**Figure 12:**
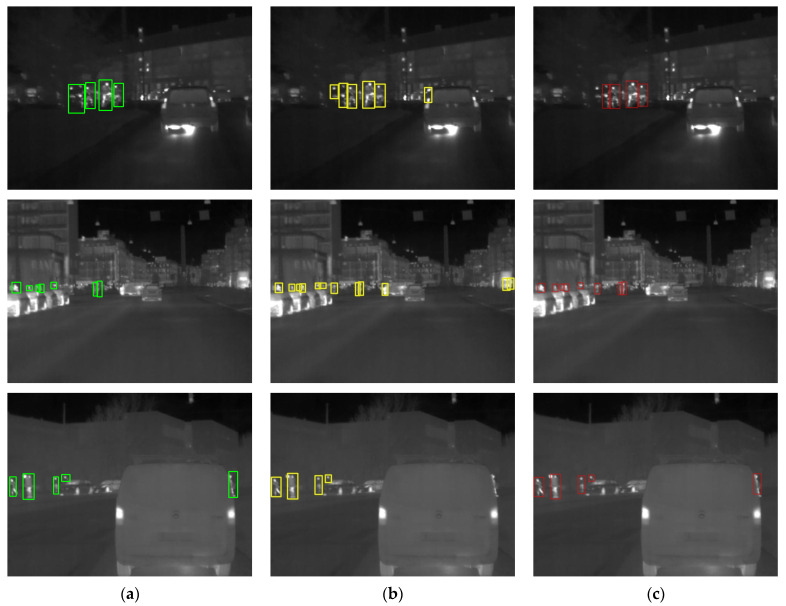
Infrared pedestrian detection instance, where the green boxes represent the pedestrians annotated in the dataset, the yellow boxes represent the detection results inferred by YOLOv5s, and the red boxes represent the detection results inferred by IPD-Net: (**a**) Infrared image with annotation; (**b**) YOLOv5s detection results; (**c**) IPD-Net detection results.

To visually and vividly demonstrate the detection capability of IPD-Net in real-life scenarios, we show a graph of the detection results on the road in Figure 12. Figure 12a shows a picture of the ground truth, Figure 12b illustrates the detection results of the YOLOv5s, and Figure 12c shows the graph of the results obtained from the IPD-Net. In complex backgrounds, YOLOv5s has many missed and false pedestrian detections, but IPD-Net has excellent performance. In addition, IPD-Net also achieves better performance when conducting small-scale pedestrian detection.

## 5. Conclusions

This paper analyses the shortcomings of existing object detection algorithms in the field of infrared pedestrian detection. To solve the problem of pedestrian detection in infrared images, the pedestrian detection algorithm IPD-Net was proposed. Firstly, an AFEM is designed to solve the problem of low pedestrian detection accuracy due to low detail and inconspicuous features of the object in infrared images. The AFEM enhances the feature extraction capability of the backbone and improves the detection performance for weak and small objects. Secondly, we designed a CA-FPN to make full use of the weak and small object information in the shallow feature map and to dig deeper into the localization information in the deep feature map. The CA-FPN makes the fusion of feature maps more balanced and improves the representation of pedestrian features by means of concatenation. Finally, we analyzed the shortcomings of the CIoU loss function in the bounding box regression process. Our proposed α-EIoU loss function has better regression performance in the infrared image pedestrian detection process.

This paper provides a solution for pedestrian detection in poor lighting and severe weather conditions, IPD-Net. The IPD-Net model improves the accuracy of pedestrian detection in infrared images. It can be deployed in industrial applications such as autonomous driving, intelligent transportation, and intelligent surveillance.

## Figures and Tables

**Table 1:** Ablation experiment.

Baseline	AFEM	CA-FPN	α-EIoU	mAP50/%	mAP(50:95)/%
√				75.7	30.3
√	√			76.2	30.5
√		√		77.9	31.6
√			√	76.7	30.7
√	√	√		78	31.5
√	√		√	77.7	31.2
√		√	√	78.9	31.9
√	√	√	√	**79.3**	**32.1**

The bolded data represent the best experimental results.

**Table 2:** Experimental results with different input image sizes.

Image Size	Method	mAP50/%	mAP(50:95)/%
320 × 320	YOLOv5s	75.7	30.3
	IPD-Net	**79.3**	**32.1**
480 × 480	YOLOv5s	82.1	33.9
	IPD-Net	**84.3**	**35.2**
640 × 640	YOLOv5s	84.4	35.5
	IPD-Net	**86.5**	**36.6**

The bolded data represent the best experimental results.

**Table 3:** Experimental comparison of IPD-Net with YOLO series algorithms.

Method	Image Size	mAP50/%	mAP(50:95)/%
YOLOv3-tiny	320	64.3	24.3
YOLOv4-tiny	320	70.4	25.4
YOLOv5s	320	75.7	30.3
YOLOX-s	320	78.4	31.3
YOLOv7	320	72.4	28.1
IPD-Net	320	**79.3**	**32.1**

The bolded data represent the best experimental results.

**Table 4:** Experimental comparison of IPD-Net with other models.

Category	Method	Image Size	mAP50/%	mAP(50:95)/%
Two-stage	Faster R-CNN	640	83.7	34.7
	Mask R-CNN [43]	640	83.4	34.8
	Cascade R-CNN [44]	640	84	36.3
	Grid R-CNN [45]	640	83.8	36.2
	CenterNet [46]	640	77.5	30.3
	Libra R-CNN [47]	640	82.9	34.8
One-stage	RetinaNet	640	77.2	31.3
	YOLOv3-tiny	640	78.8	32.1
	YOLOv4-tiny	640	79.1	32.6
	YOLOv5s	640	84.4	35.5
	EfficientDet-D3	640	81.4	33.5
	YOLOX-s	640	82.7	34
	YOLOv7	640	84	34.4
	IPD-Net	640	**86.5**	**36.6**

The bolded data represent the best experimental results.

## Data Availability

Not applicable.
